# Recurrent activating *STAT5B* N642H mutation in myeloid neoplasms with eosinophilia

**DOI:** 10.1038/s41375-018-0342-3

**Published:** 2018-12-20

**Authors:** Nicholas C. P. Cross, Yvette Hoade, William J. Tapper, Gonzalo Carreno-Tarragona, Tiziana Fanelli, Mohamad Jawhar, Nicole Naumann, Iwo Pieniak, Johannes Lübke, Sahra Ali, Kaljit Bhuller, Sonja Burgstaller, Catherine Cargo, Jamie Cavenagh, Andrew S. Duncombe, Emma Das-Gupta, Paul Evans, Peter Forsyth, Philip George, Charlotte Grimley, Fergus Jack, Laura Munro, Varun Mehra, Kavita Patel, Ali Rismani, Gabriela Sciuccati, Rowena Thomas-Dewing, Patrick Thornton, Andres Virchis, Simon Watt, Louise Wallis, Alastair Whiteway, Kris Zegocki, Barbara J. Bain, Andreas Reiter, Andrew Chase

**Affiliations:** 1 Faculty of Medicine, University of Southampton, Southampton, UK; 2 Wessex Regional Genetics Laboratory, Salisbury NHS Foundation Trust, Salisbury, UK; 3 Center Research and Innovation of Myeloproliferative Neoplasms, AOU Careggi, University of Florence, Firenze, Italy; 4 University Hospital Mannheim, Heidelberg University, Mannheim, Germany; 5 Hull & East Yorkshire Hospitals NHS Trust, Hull, UK; 6 University Hospitals of Leicester NHS Trust, Leicester, UK; 7 Klinikum Wels-Grieskirchen, Wels, Austria; 8 HMDS, St. James’s University Hospital, Leeds, UK; 9 St. Bartholomew’s Hospital, London, UK; 10 University Hospitals Southampton, Southampton, UK; 11 Nottingham University Hospitals NHS Trust, Nottingham, UK; 12 Raigmore Hospital, Inverness, UK; 13 Poole Hospital NHS Trust, Poole, UK; 14 York Teaching Hospital NHS Trust, York, UK; 15 King’s College Hospital, London, UK; 16 Mid Yorkshire Hospitals NHS Trust, Wakefield, UK; 17 Whittington Health & University College London Hospitals, London, UK; 18 Hospital de Pediatria “Prof. Dr. Garrahan”, Buenos Aires, Argentina; 19 Salford Royal Hospital, Salford, UK; 20 Beaumont and Connolly Hospitals, Dublin, Ireland; 21 Royal Free London, Barnet Hospital, Wellhouse Lane, Barnet, UK; 22 Manchester University NHS FT, Manchester, UK; 23 Royal Bournemouth Hospital, Bournemouth, UK; 24 Southmead Hospital, Bristol, UK; 25 Whipps Cross University Hospital, London, UK; 26 St. Mary’s Hospital, London, UK

**Keywords:** Cancer genetics, Oncogenesis

## Abstract

Determining the underlying cause of persistent eosinophilia is important for effective clinical management but remains a diagnostic challenge in many cases. We identified *STAT5B* N642H, an established oncogenic mutation, in 27/1715 (1.6%) cases referred for investigation of eosinophilia. Of the 27 mutated cases, a working diagnosis of hypereosinophilic syndrome (HES; *n* = 7) or a myeloid neoplasm with eosinophilia (*n* = 20) had been made prior to the detection of *STAT5B* N642H. Myeloid panel analysis identified a median of 2 additional mutated genes (range 0–4) with 4 cases having *STAT5B* N642H as a sole abnormality. *STAT5B* N642H was absent in cultured T cells of 4/4 positive cases. Individuals with *SF3B1* mutations (9/27; 33%) or *STAT5B* N642H as a sole abnormality had a markedly better overall survival compared to cases with other additional mutations (median 65 months vs. 14 months; hazard ratio = 8.1; *P* < 0.001). The overall survival of *STAT5B*-mutated HES cases was only 30 months, suggesting that these cases should be reclassified as chronic eosinophilic leukemia, not otherwise specified (CEL-NOS). The finding of *STAT5B* N642H as a recurrent mutation in myeloid neoplasia with eosinophilia provides a new diagnostic and prognostic marker as well as a potential target for therapy.

## Introduction

Eosinophilia, defined as an elevation of the peripheral blood (PB) eosinophil count above 0.5 × 10^9^/L, is conventionally divided into three main categories: primary, secondary (reactive) and idiopathic. Primary eosinophilia is a clonal hematologic disorder in which the eosinophils form part of the neoplastic clone. Secondary, non-clonal eosinophilia may be driven by a wide range of underlying conditions including allergic disorders, autoimmunity, infectious diseases, lymphoproliferative disorders, solid tumours, drug reactions and other conditions. Idiopathic eosinophilia is a diagnosis of exclusion when no primary or secondary cause can be identified [1].

Clonal eosinophilia is seen in the context of a myeloid neoplasm and particularly the World Health Organisation (WHO)-defined entities ‘chronic eosinophilic leukemia, not otherwise specified’ (CEL-NOS) and ‘myeloid and lymphoid neoplasms with rearrangement of *PDGFRA*, *PDGFRB* or *FGFR1* or with *PCM1-JAK2*, *ETV6-JAK2* or *BCR-JAK2*’ (MLN-eo). Clonal eosinophilia may also be associated with other WHO subtypes of myeloproliferative neoplasm (MPN-eo) or myelodysplastic/myeloproliferative neoplasm (MDS/MPN-eo) [1–3].

Identifying the underlying cause of eosinophilia is important for patient management but can be challenging in the absence of an overt myeloid neoplasm or discernible secondary cause. Primary eosinophilia is strongly associated with constitutively activated tyrosine kinase (TK) signalling, and to date more than 70 TK fusion genes have been identified in myeloid neoplasms as a consequence of reciprocal translocations or other genomic rearrangements [3]. Identification of these fusions usually confirms a specific diagnosis and is an indication for targeted therapy. Notably, imatinib induces rapid and durable complete clinical, hematologic and molecular remissions in >90% of patients with a *PDGFRA* or *PDGFRB* fusion gene, conferring excellent progression-free and overall survival (OS) [4, 5]. Fusions involving *JAK2*, *FGFR1* or *FLT3* are associated with a more aggressive clinical course and may be responsive to other small molecule inhibitors [6–8]. Some TK-fusion negative eosinophilia cases test positive for *KIT* D816V or *JAK2* V617F, whereas others have mutations in a range of genes associated with myeloid neoplasms such as *TET2*, *ASXL1*, *EZH2* or *SETBP1* [9–11]. It has been suggested that a rapid and durable response to corticosteroids is uncommon in cases with primary eosinophilia and instead points towards a diagnosis of secondary eosinophilia, if that is not already apparent [12].

In this study, we have used genomic approaches to focus on the identification of novel somatic abnormalities in patients with suspected primary eosinophilia. We have identified a recurrent somatically acquired point mutation in *STAT5B* leading to an N642H substitution in several cases. This mutation is known to activate STAT5B but was previously thought to be restricted to lymphoproliferative disorders.

## Methods

### Patients and samples

Our study included samples from individuals referred for routine diagnostic analysis of persistent unexplained eosinophilia and/or patients diagnosed with MPN or MDS/MPN with eosinophilia according to standard morphologic, hematologic and laboratory criteria [2]. Cases that tested positive for *KIT* D816V, *FIP1L1-PDGFRA* or other recognised TK fusion genes were excluded. The study design adhered to the tenets of the Declaration of Helsinki and was approved by the National Research Ethics Service (UK) Committee South West and the institutional review board of the Medical Faculty of Mannheim, Heidelberg University as part of the ‘German Registry on Disorders of Eosinophils and Mast Cells’. DNA or RNA was extracted from total PB or bone marrow (BM) leukocytes using standard procedures. T-cells were selected using CD3 microbeads (Miltenyi Biotech, Cologne, Germany) and stimulated to divide by co-cultivation with CD2/CD3/CD28 in the presence of interleukin-2 (T-cell activation/expansion kit; Miltenyi Biotech).

### RNAseq analysis

RNAseq data for the initial cohort of 14 cases is available at ArrayExpress (www.ebi.ac.uk/arrayexpress; accession number E-MTAB-7492). RNAseq data was analysed for point mutations using an in-house pipeline. Briefly, raw RNAseq data in fastq format were aligned to the reference genome (human_g1k_v37) using STAR aligner and a two-step process. In the first step, splice junctions were detected by an initial alignment of the fastq files. In the second step, final alignments were determined using the splice junctions as a guide [13]. After alignment the bam files were sorted, de-duplicated and read groups were added using Picard (http://broadinstitute.github.io/picard). In preparation for variant calling, GATK was used to hard-clip intronic sequences (SplitNCigarReads), reassign mapping qualities (ReassignOneMappingQuality) and recalibrate base quality scores (BaseRecalibrator). Variants were called using GATK HaplotypeCaller and ignoring soft clipped bases, which minimises false positive and false negative calls. A final set of high-quality variants in variant call format (VCF) was produced by selecting variants with a phred-scaled confidence threshold of 20 and excluding variants with low depth corrected quality scores (QD < 2), significant strand bias (FS > 30) or SNP clusters of 3 or more SNPs within 35 base pairs. ANNOVAR [14] was used to annotate variants with respect to genes, databases of known polymorphisms (Exome Aggregation Consortium, 1000 genomes, exome sequencing project, dbSNP), and pathogenicity estimates (avsift, polyphen, etc.). A shortlist of potentially relevant variants was created by applying additional filters, which excluded non-coding variants, synonymous SNVs and variants with an alternate allele frequency greater than 1% in public databases of known variation: 1000 genomes (1000G; www.ncbi.nlm.nih.gov/variation/tools/1000genomes/), exome sequencing project (ESP; http://evs.gs.washington.edu/EVS/) and exome aggregation consortium (ExAC; http://exac.broadinstitute.org/). Variants passing these filters were further highlighted if they were located in a manually curated list of candidate genes (*n* = 581 genes), annotated as pathogenic in Clinvar, overlapped with a somatic mutation in hematopoietic and lymphoid tissue in COSMIC (https://cancer.sanger.ac.uk/cosmic) or were recurrently mutated.

### Amplification-refractory mutation system (ARMS) test for STAT5B N642H

A tetra-primer ARMS assay [15] was designed using http://primer1.soton.ac.uk/primer1.html with inner primers to specifically amplify the normal and mutant *STAT5B* alleles and outer primers to produce a positive control *STAT5B* band for each reaction. PCR primers were: forward outer (FO), 5′-CGATCAGGAAACACGTAGATAAGGTAATT-3; reverse outer (RO), 5′-AAATGGAGATTTCTATTGGAGCCATTAT-3; forward wild-type specific (Fwt), 5′-TCTCTGGTGGTAAAAGGCATCAGGTT-3; reverse mutant-specific (Rmt), 5′-TTATTGATCTAGAGGAAAGAATGTTTTAGC-3. The FO and RO were used at a final concentration of 0.5 μM whereas the inner primers Fwt and Rmt were used at 2.5 μM. Amplification reactions were performed using AmpliTaq Gold DNA polymerase, 25 ng genomic DNA at an annealing temperature of 60 °C for 35 cycles. Cases showing mutant bands after agarose gel electrophoresis were confirmed by independent amplification followed by Sanger sequencing.

### Other mutational analysis

Sanger sequencing was used to screen for other *STAT* gene mutations using primers designed to amplify from cDNA: *STAT3* exons 20–22 (5′-GGGCCATCTTGAGCACTAAG-3′ and 5′-CACAGATAAACTTGGTCTTCAGG-3′); *STAT5A* exons 16–17 (5′-GGACCTTCTTGTTGCGCTTT-3′ and 5′-GGCGGTCAGGAAACACATAG-3′) and *STAT5B* exons 16–17 (5′-AGTGACTCAGAAATTGGCGG-3′ and 5′-GGCCTGGTCCATGTACGT-3′). ARMS assays were designed for JAK3 V722I and SOCS1 Q201H. For JAK3 V722I, a standard tetra primer assay was designed using PCR primers: forward outer (FO), 5′-CAATAGACCCACCCCAATCTCCCCAGAC-3′; reverse outer (RO), 5′-GCAAGGAAGTGGATCCCTGATCCCACTT-3′; reverse wild-type-specific (Rwt), 5′-GCCACGGTCTGGGAAGTGTTTAGTGtCG-3′; forward mutant-specific (Fmt), 5′-ATCCAGGGCACTGATGGGCATGGTTAT-3′. FO, RO, Rwt and Fmt primers were all used at a final concentration of 0.5 μM in PCR reactions using AmpliTaq Gold DNA polymerase, 25 ng genomic DNA and an annealing temperature of 68 °C for 35 cycles. Design of a tetra primer assay for SOCS1 Q201H proved to be difficult and therefore we used a simplified mutation-specific assay with primers forward (F) 5′-CCAGGAGGGGGAGGACCCCCTCAAGAGG-3′ and reverse mutant-specific (Rmt), 5′-CGCGACTACCTGAGCTCCTTCCCCTTCGAC-3′ at an annealing temperature of 70 °C for 35 cycles. All ARMS assays were validated with control samples, and positive/negative controls were included on each run. The Illumina Trusight Myeloid Sequencing Panel (Illumina, San Diego, CA) was used to screen *STAT5B*-mutated samples for additional somatic mutations. Samples were processed according to the manufacturer’s protocol, run on an Illumina Miseq and results interpreted using Alissa Interpret (Agilent, Cheadle, UK) using a variant allele frequency (vaf) cut off of ≥5%.

### Colony growth and sequencing

Mononuclear cells from cryopreserved or fresh primary cells were grown in methylcellulose with cytokines, without erythropoietin (Methocult H4035 Optimum), no EPO, Stemcell Technologies (Vancouver, BC, Canada). DNA was prepared for sequencing using the PicoPlex whole genome amplification kit (Rubicon Genomics Inc., Ann Arbor, MI). Colonies were first plucked into 80 µl phosphate buffered saline, spun down and resuspended in 2.5 µl of PicoPlex cell extraction buffer followed by DNA extraction and amplification according to the manufacturer’s instructions. The DNA was then cleaned up using the QIAquick PCR purification kit (Qiagen, Hilden Germany) prior to amplification and Sanger sequencing using primers designed to target specific mutations.

### Statistical analysis

OS probabilities were estimated using the Kaplan–Meier method and compared by the log-rank (Mantel–Cox) test using SPSS v25 (IBM Corporation, Armonk, NY, USA). OS was defined as the time between diagnosis and the date of death or last contact.

## Results

### STAT5B N642H mutation identified by RNAseq

We previously described RNA-seq analysis to search for cryptic fusion genes in 14 patients with MPN-eo or idiopathic hypereosinophilia with a normal karyotype [6]. Reanalysis of these data for possible point mutations identified 10 candidate variants in 6 cases (Table 1). No variants were seen in 8 cases, including the 2 previously reported to have *DIAPH1-PDGFRB* or *ZMYM2-FLT3* fusions [6]. Two of the 6 cases had known myeloid driver mutations: *JAK2* V617F in case E166 and *SF3B1* K666N in case E11076. Of the other variants, *STAT5B* N642H (NM_012448: c.A1924C) was seen in two cases (E11076 and E11493) and stood out as this is a known driver mutation in lymphoproliferative disorders [16]. Furthermore, this mutation was recently reported in the T-cells and other cell lineages of two young children with eosinophilia [17].

**Table 1:** Candidate variants identified by RNAseq analysis

Case	Gene	Refseq	cDNA	Protein	vaf
E166	*JAK2*	NM_004972	c.G1849T	p.V617F	1.00
E166	*SOCS1*	NM_003745	c.G630C	p.Q210H	0.48
E11072	*RPS19*	NM_001022	c.C164T	p.T55M	0.48
E11072	*SOCS1*	NM_003745	c.G630C	p.Q210H	0.86
E11075	*PRF1*	NM_001083116	c.A755G	p.N252S	0.39
E11076	*SF3B1*	NM_012433	c.G1998T	p.K666N	0.32
E11076	*PRF1*	NM_001083116	c.A755G	p.N252S	0.67
E11076	*STAT5B*	NM_012448	c.A1924C	p.N642H	0.43
E11493	*STAT5B*	NM_012448	c.A1924C	p.N642H	0.47
E11500	*JAK3*	NM_000215	c.G2164A	p.V722I	0.56

### Identification of additional cases with STAT5B mutations

**Fig. 1:**
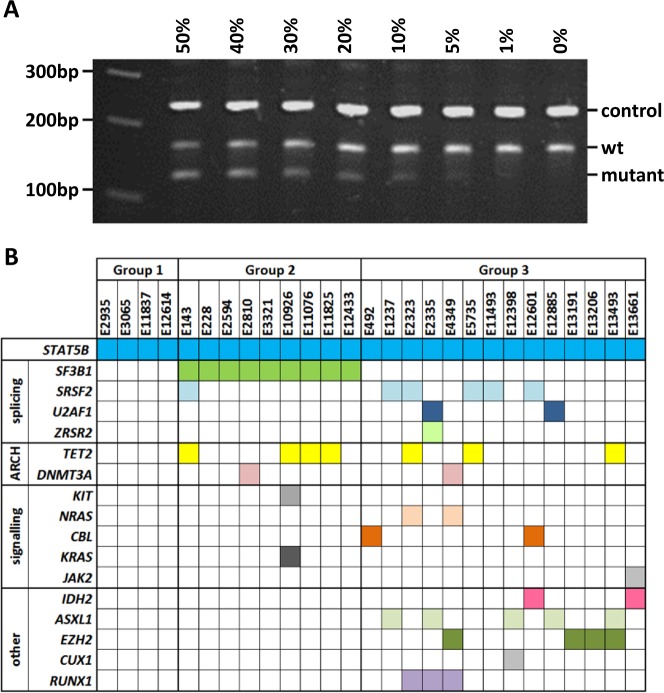
**a** ARMS assay for *STAT5B* N642H. Heterozygotes have 3 bands—control band (243 bp), wild type (wt) specific band (166 bp) and mutant allele specific band (124 bp). Using serial dilutions of DNA from a mutated case we estimated that the assay can detect *STAT5B* N642H at a variant allele frequency of 10%. **b** Summary of mutations for all 27 *STAT5B* N642H mutated cases as determined by ARMS PCR, Sanger sequencing and myeloid panel analysis. Group 1 are cases with no additional mutated genes, group 2 have additional mutated genes that include *SF3B1* and group 3 have additional mutated genes that do not include *SF3B1*. ARCH = genes strongly associated with age-related clonal hematopoiesis. Full details of additional mutations are given in Supplementary Table 1

We used an ARMS assay to rapidly screen additional retrospective cases for *STAT5B* N642H (Fig. 1a). In an initial screen, we found that 2/30 cases with suspected MPN-eo tested positive compared to 0/74 cases with other chronic myeloid neoplasms. Subsequent screening focused only on cases referred for investigation of eosinophilia: of 1671 cases screened, 23 further cases tested positive. Overall, therefore, we found that 27/1715 (1.6%) eosinophilia patients harboured *STAT5B* N642H. For 25/26 cases confirmed by Sanger sequencing, the mutation-specific peak was at a similar height or somewhat lower than the wild type peak, suggesting that the majority of leukocytes were heterozygous for *STAT5B* N642H. In one sample (E492) only the *STAT5B* mutant peak was seen suggesting the dominance of a homozygous or hemizygous mutant clone (Supplementary Figure 1). In 4/4 cases tested, the *STAT5B* mutant clone was not detected in cultured T-cells confirming that it was acquired somatically (Supplementary Figure 2).

### Other STAT mutations

Other somatic *STAT5B and STAT3* mutations have been identified in lymphoproliferative disorders. To test if these mutations might also be associated with persistent eosinophilia, we sequenced known mutation hotspot regions of *STAT3* and *STAT5B* in 153 cases but no variants were seen apart from *STAT5B* N642H. No human mutations have been reported in *STAT5A* but due to the high homology with *STAT5B* we also sequenced part of this gene, but again no variants were found (149 cases).

### Additional mutations in STAT5B-mutated cases

We screened all 27 *STAT5B* N642H cases for mutations in other genes associated with myeloid neoplasms (Fig. 1b). Overall, mutations were seen in a median of two additional genes (range 0–4), with 4 cases showing no additional variants (Group 1). Of note, 9 cases had *SF3B1* mutations (Group 2) of which 4 were detected as sole additional abnormalities, 3 were seen in combination with single mutations in *TET2* or *DNMT3A* and 2 had additional mutations in genes encoding signalling or splicing components. Fourteen cases did not have *SF3B1* mutations but instead had a diverse range of epigenetic, signalling, transcription factor and other splicing mutations with no clear patterns emerging in terms of co-mutated genes (Group 3). All additional mutations are detailed in Supplementary Table 1.

### Clinical features associated with STAT5B N642H and other mutations

**Fig. 2:**
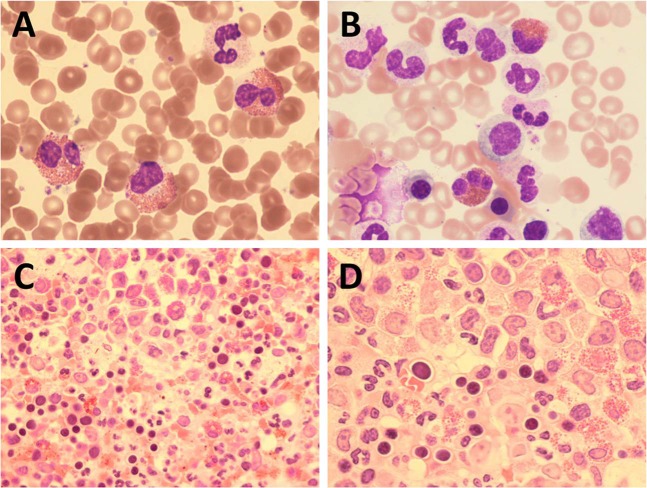
Peripheral blood film (**a**), bone marrow smear (**b**) and bone marrow trephine biopsy section at ×20 (**c**) and ×100 (**d**). The blood film showed 40% neutrophils, 28% eosinophils, 4% basophils, 12% lymphocytes and 7% monocytes. The eosinophils were morphologically close to normal with only a very minor degree of vacuolation and hypogranularity but there was an increase in non-lobated forms. Bone marrow cellularity and megakaryocyte numbers were increased. The increased cellularity was due to an increase in all three granulocyte lineages (neutrophils, basophils and eosinophils). The trephine biopsy sections show hypercellularity, disorganisation and an increase in cells of neutrophil and eosinophil lineages. Reticulin was not increased

The clinical features for all cases are summarised in Table 2. There was a preponderance of males (19 males, 8 females) and the median age was 70 (range 7–89; *n* = 27) with a median eosinophil count at the presentation of 6 × 10^9^/L (range 0.5–27; *n* = 26). In most cases eosinophilia was apparent at diagnosis but in 3 cases this was acquired during the course of myelodysplastic syndrome (MDS; cases E2594 and E11837) or MPN (case E13661). Basophilia was noted in several cases. A working diagnosis of idiopathic hypereosinophilic syndrome (HES) had been made in 7 cases prior to the finding of *STAT5B* N642H and CEL-NOS in 2 cases, supported by the finding of an additional chromosome 8 by cytogenetic analysis in both cases. The Kaplan–Meier estimate for OS of *STAT5B-*mutated HES cases was only 30 months, which is very short compared to published series [11], and suggests that these cases should be reclassified as CEL-NOS [10]. The remaining 18 cases had been diagnosed with another myeloid neoplasm, most commonly a subtype of MDS/MPN (*n* = 11). The PB and BM from a representative case with CEL-NOS and *STAT5B* N642H as a sole abnormality are shown in Fig. 2.

**Table 2:** Clinical summary of *STAT5B* N642H mutant cases

Case	Sex	Age (y)	Status (m)	wcc	plt	Hb	eos	Initial diagnosis	MG
E143	M	69	D (107)	16	280	100	1.5	aCML	2
E228	M	65	n/a	n/a	n/a	n/a	n/a	HES	2
E492	F	7	D (13)	30	343	147	11	Myeloid sarcoma	3
E1237	M	89	D (12)	15	488	148	6	MPN-U	3
E2323	M	70	D (12)	61	107	101	10	CMML	3
E2335	M	63	D (9)	28	113	158	0.7	MDS/MPN	3
E2594	M	58	D (14)	14	128	81	4	MDS-SLD; eosinophilia at 3 years	2
E2810	M	81	D (7)	18	409	94	10	MPN-U	2
E2935	F	10	D (48)	47	157	110	27	HES	1
E3065	F	82	D (28)	25	150	100	2	CMML + MM	1
E3321	M	86	D (45)	21	n/a	152	6	HES	2
E4349	M	61	D (12)	15	16	132	5	CMML	3
E5735	M	68	D (31)	12	106	111	5	HES	3
E10926	M	70	D (16)	142	111	73	1.4	SM-AHN	2
E11076	M	72	A (68)	21	556	149	6	MDS/MPN-U	2
E11493	M	76	D (23)	42	275	92	19	MDS/MPN-U	3
E11825	F	61	A (61)	9	438	108	1.2	MDS/MPN-RS-T	2^a^
E11837	M	72	D (14)	11	120	156	7	MDS-MLD; eosinophilia at 3 years	1
E12398	M	77	D (15)	24	34	140	7	HES	3^a^
E12433	F	51	A (35)	19	151	90	3	MDS/MPN-U	2
E12601	F	64	D (14)	28	267	116	10	HES	3
E12614	F	15	A (48)	28	655	133	4	CEL-NOS	1^a^
E12885	F	75	D (23)	24	238	110	3	MDS/MPN	3^a^
E13191	M	83	A (9)	29	401	148	13	HES	3
E13206	M	85	D (4)	39	229	142	20	CEL-NOS	3
E13493	M	70	D (9)	17	195	65	6	MDS/MPN-U	3
E13661	M	42	A (60)	12	267	125	0.5	PV; eosinophilia at 25 years	3

Sex: male (M) or female (F); Age in years (y) at presentation; Status: dead (D) or alive (A) at specified number of months (m) after presentation or first detection of eosinophilia (E2594, E11837, E13661); white cell count (wcc), platelets (plt) and eosinophils (eos) ×10^9^/L at diagnosis or first detection of eosinophilia; haemoglobin (Hb) in g/L; diagnosis: atypical chronic myeloid leukemia (aCML), hypereosinophilic syndrome (HES), myeloproliferative neoplasm, unclassifiable (MPN-U); myelodysplastic/myeloproliferative neoplasm (MDS/MPN), myelodysplastic syndrome with single lineage dysplasia (MDS-SLD), chronic myelomonocytic leukemia (CMML), multiple myeloma (MM), systemic mastocytosis with associated hematological neoplasm (SM-AHN), MDS/MPN, unclassifiable (MDS/MPN-U), MDS/MPN with ring sideroblasts and thrombocytosis (MDS/MPN-RS-T), MDS with multilineage dysplasia (MDS-MLD), chronic eosinophilic leukemia, not otherwise specified (CEL-NOS), polycythemia vera (PV); molecular group (MG): 1 = no additional mutations; 2 = *SF3B1* mutated; 3 = additional mutations but *SF3B1* unmutated; not available (n/a)

^a^Indicates the 4 cases for whom *STAT5B* N642H was absent in cultured T-cells

**Fig. 3:**
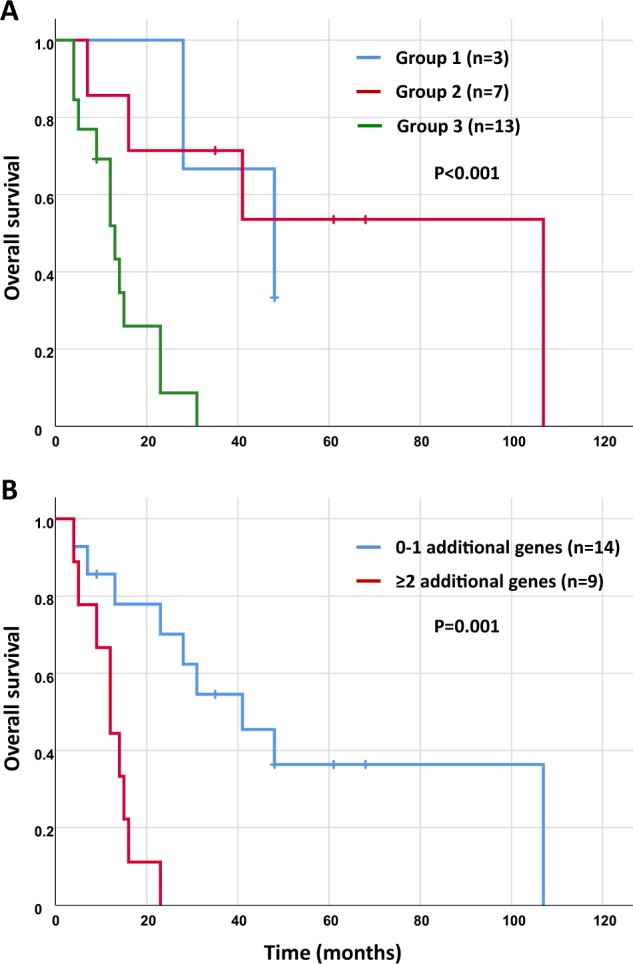
Kaplan–Meier plots showing overall survival (OS) estimates for the *STAT5B* N642H mutated cases who had eosinophilia at diagnosis and had follow up data available (*n* = 23). **a** Comparison of the 3 molecular groups shows that cases in group 3 (those with additional mutated genes that do not include *SF3B1*) have an inferior OS compared to all other cases (median 14 months vs. 65 months; *P* < 0.0004). **b** OS for cases with mutations in 2 or more additional genes (excluding *DNMT3A* and *TET2*) was significantly worse than that for cases with 0 or 1 additional mutations (median 18 months vs. 50 months; *P* = 0.001). Of the 9 cases with mutations in ≥2 additional genes, 8 were in group 3

There was no obvious correlation between the clinical diagnosis and the molecular classification described above. For example, the 7 cases initially diagnosed with HES were split between molecular group 1 (*n* = 1), group 2 (*n* = 2) and group 3 (*n* = 4) and the 9 cases with *SF3B1* mutations had been diagnosed with 7 different entities, including HES (*n* = 2) and 6 WHO-defined subtypes of myeloid neoplasms. There was, however, a clear correlation between molecular features and outcome. Focusing on patients with eosinophilia at diagnosis, the OS for cases in group 3 was markedly inferior to cases in groups 1 and 2 [median 14 months vs. 65 months; hazard ratio (HR) = 8.1 (95% CI: 1.9–23); *P* < 0.0004, Fig. 3a]. By contrast age, gender and white cell count were not significantly associated with OS in this relatively small group, whereas eosinophil count approached significance (≤6 × 10^9^/L, 49 months vs. >6 × 10^9^/L, 17 months; *P* = 0.06). No significant effect of mutation number was seen when all mutations were considered but, interestingly, when genes strongly associated with age-related clonal hematopoiesis (i.e. *DNMT3A* and *TET2*) were excluded from the analysis, the OS for cases with mutations in 2 or more additional genes was significantly worse than that of cases with 0 or 1 additional mutations [median 18 months vs. 50 months; HR = 6.5 (95% CI: 2.1–30) *P* = 0.001, Fig. 3b].

As for therapy, 5 patients were treated with imatinib for at least 2 months but none responded. One patient with *KIT* D816V-positive systemic mastocytosis with associated hematologic neoplasm (SM-AHN) responded to midostaurin but the response was lost after 9 months. Some patients showed clinical improvement and reduction in eosinophil counts with corticosteroids but in most cases this was partial and/or transient.

### Clonal hierarchy

**Fig. 4:**
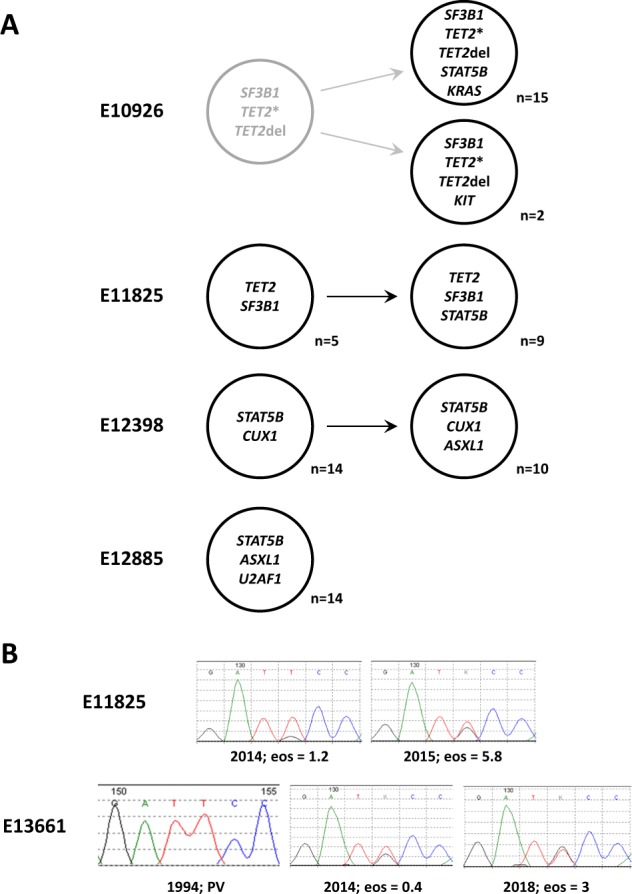
**a** Clonal hierarchy for 4 patients. The numbers indicate the number of colonies with that genotype, e.g. for case E11825, 5 colonies were mutant for *TET2* and *SF3B1* but not *STAT5B* and 9 colonies were mutant for all 3 genes. Light grey indicates an inferred ancestral clone. **b** Sequential analysis of two cases with increasing eosinophil counts (values indicated are ×10^9^/L). Case E11825 had an elevated eosinophil count at diagnosis, with eosinophilia increasing over time co-incident with an increase in *STAT5B* N642H vaf. Case E13661 was diagnosed initially with PV in 1988 and *STAT5B* N642H was not detected in a sample taken 6 years later. Slightly elevated eosinophil counts were first noted in 2014, and a sample from this time was positive for *STAT5B* N642H. The mutant vaf and eosinophil counts increased over the next 4 years

To understand if the acquisition of *STAT5B* N642H is an early or late event in the development of myeloid neoplasia, we genotyped myeloid colonies grown from 4 cases with multiple mutations. For case E10926, 6 mutations in 5 genes had been identified by bulk analysis. By colony analysis, we found a major clone with 5 mutations that included *STAT5B* N642H and *KRAS* V14I but not *KIT* D816V, and a minor clone that included *KIT* D816V but not *STAT5B* N642H or *KRAS* V14I. All colonies tested positive for *SF3B1* and two independent *TET2* mutations and thus branching evolution can be inferred with *STAT5B* N642H acquired in a later subclone. For 2 of the other cases, linear evolution was apparent with *STAT5B* and *ASXL1* mutations acquired as late events. The fourth case was uninformative with all colonies positive for the 3 mutations detected on bulk analysis (Fig. 4a). For two cases with increasing eosinophil counts, the *STAT5B* N642H vaf, as estimated by Sanger sequencing, increased over time (Fig. 4b).

### Assessment of other variants identified by RNAseq

Other recurrent mutations identified by RNAseq were also considered. Both *PRF1* N252S and *SOCS1* Q210H were identified in two individuals (Table 1). *PRF1* encodes perforin 1, a gene associated with familial hemophagocytic lymphohistiocytosis. However, the N252S variant has been shown to be a non-functional rare single nucleotide polymorphism (SNP) and was therefore not considered further [18]. *SOCS1* encodes suppressor of cytokine signalling 1, which takes part in a negative feedback loop to attenuate cytokine signalling and therefore has obvious potential relevance to myeloid neoplasia. The status of the Q210H variant is unclear: it is seen at a frequency of 0.2–0.6% in the 1000G, ESP and ExAC control datasets, as are some known somatic driver mutations such as *JAK2* V617F. For one case, we tested buccal cells and found the variant was constitutional (data not shown). To test the possibility that Q210H might be more widespread in myeloid neoplasia, we tested cases referred for investigation of eosinophilia (*n* = 151), mastocytosis (*n* = 75), MDS/MPN (*n* = 54) and healthy controls (*n* = 88). Just 3 (1%) cases tested positive (one with mastocytosis, 2 with MDS/MPN) and none of the controls (*P* = 0.99; Fisher’s exact test) suggesting that this variant is an irrelevant, rare polymorphism that happened to be present in 2 of the cases analysed by RNAseq. *JAK3* V722I was seen in a single case and has been reported previously as an activating mutation [19] but is also present at 0.4–1% in control datasets and its pathogenicity has been disputed. We found that 4/307 cases with suspected myeloid neoplasia tested positive for *JAK3* V722I (one of which also had the mutation in cultured T-cells) as well as 3/88 normal controls (*P* = 0.19), again suggesting it is an irrelevant polymorphism. Similarly, *RPS19* T55M is also believed to be a polymorphism [20].

## Discussion

STAT5 is a key component of cytokine-induced signal transduction cascades, and a critical downstream mediator of transformation by oncogenic TKs such as BCR-ABL1, JAK2 V617F, FLT3-ITD and ZNF198-FGFR1 [21–25]. STAT5 is encoded by two different genes, *STAT5A* and *STAT5B*, located closely together at chromosome 17q11.2 that encode proteins with >90% amino acid identity and largely redundant functions. Nevertheless, targeted disruption of *STAT5A* and *STAT5B* gives rise to distinct phenotypes in mice and only *STAT5B* has been reported to be a target of mutations in cancer [26].

*STAT5A* N642H was initially identified as a constitutively activating mutation in a random ex vivo mutagenesis screen [27]. The corresponding mutation in *STAT5B* was subsequently identified as a somatically acquired driver mutation in 1–37% of patients with various lymphoid neoplasms including large granular lymphocytic leukemia [16], paediatric T-cell acute lymphoblastic leukemia [28], T-cell prolymphocytic leukemia [29], γδ-T-cell lymphoma [30] and two cases of lymphocyte-driven early onset nonclonal eosinophilia with urticaria, dermatitis and other features [17]. The most frequent mutation seen in these T-cell disorders is *STAT5B* N642H, but other mutations are also seen, specifically *STAT5B* Y665F and *STAT3* Y640F, N647I and D661V/Y [31]. Isolated reports have identified single cases of myeloid neoplasms, specifically chronic neutrophilic leukemia [32] and MLN-eo [33], that tested positive for *STAT5B* N642H as well as 2 cases that developed clonal hematopoiesis following aplastic anaemia [34]. Our findings are the first to identify *STAT5B* N642H as a recurrent mutation in myeloid neoplasms with eosinophilia.

We demonstrated the absence of *STAT5B* N642H in cultured T-cells from 4 cases, one of whom (case E12614) was in molecular group 1, i.e. had no additional mutations. All 4 group 1 cases, and indeed most cases in this study, had *STAT5B* vafs which suggested the great majority of leukocytes were heterozygously mutated (Supplementary Figure 1). Although we cannot exclude the possibility that some T-cell subsets might be part of the mutant clone, our data strongly suggest that *STAT5B* N642H drives primary eosinophilia irrespective of the presence or absence of additional mutations.

Cytokine stimulation results in phosphorylation of STAT5B by receptor or non-receptor TKs. Dimerization of tyrosine phosphorylated (pY) STAT5B is mediated by trans-SH2 domain/phosphotyrosine binding, and the dimers then translocate to the nucleus and activate transcription of target genes [26]. N642 is located within the SH2 domain of STAT5B, close to the phosphotyrosine-binding loop. Rather than being constitutively active, the N642H mutant shows prolonged pY-STAT5 levels upon cytokine stimulation due to greatly enhanced stability of N642H homodimers [30]. Transgenic expression of *STAT5B* N642H under the control of the *Vav1* promoter (which is believed to be active in all hematopoietic cell types, including stem cells) resulted in transplantable CD8+ T-cell neoplasia. Although eosinophilia was not noted, there was a mild expansion of mature and immature myeloid lineage cells [35]. The apparent absence of myeloid neoplasia with eosinophilia in this mouse model may be the result of a number of factors, including the precise expression of *STAT5B*, the effect of other somatic mutations, the cell type in which *STAT5B* N642H arises and the rapid onset of T-cell neoplasia.

We observed a striking influence of additional mutations on patients with *STAT5B* N642H that mirrors established associations in related myeloid disorders, particularly the relatively good prognostic influence of *SF3B1* mutations and the adverse influence of multiple mutations, both of which have been described in MDS [36, 37]. In addition, only one case with an *SF3B1* mutation tested positive for one or more mutations in *SRSF2*, *ASXL1* and *RUNX1* (S/A/R), known to be an adverse prognostic factor in advanced SM [38], whereas 10/14 cases in group 3 were S/A/R positive (*P* = 0.009; Fisher’s exact test).

The combination of *JAK2* V617F and *SF3B1* mutations is associated with MDS/MPN with ring sideroblasts and thrombocytosis [39]. In our series, the working diagnosis of patients with *STAT5B* N642H and *SF3B1* mutations ranged from HES to MDS/MPN (Table 2). Stored material from most cases was not available for central morphological review but we envisage that prospective analysis of new cases will help to define more accurately the features associated with *STAT5B* N642H in the presence or absence of *SF3B1* mutations.

Previous studies have shown that STAT5A is required for eosinophil differentiation of cord blood-derived CD34+ cells [40]. Our findings, however, suggest that *STAT5B* N642H may be a driver of eosinophilia. First, in most of our cases eosinophilia was apparent at diagnosis and the level of *STAT5B* N642H as assessed by Sanger sequencing indicated that the mutation was present in the majority of cells. In 4 of these cases *STAT5B* N642H was detected as a sole abnormality, although we cannot exclude the possibility of mutations in genes not covered by the myeloid panel. Second, analysis of 2 cases with increasing eosinophil counts showed an increase in the *STAT5B* N642H vaf. Three additional cases acquired eosinophilia during the course of their disease but unfortunately samples were not available for analysis from the pre-eosinophilia phase.

The finding that STAT5B N642H shows prolonged activation following cytokine signalling suggests that targeting upstream TKs may ameliorate the activity of mutant STAT5B. Indeed, the T-cell neoplasms induced by transgenic *STAT5B* N642H was markedly suppressed by JAK1/2 inhibition [35]. Furthermore, a small molecule inhibitor of STAT5B dimerization has been shown to inhibit the growth of *FLT3*-ITD positive AML cells [41]. Further studies will be required to determine if myeloid disorders associated with *STAT5B* N642H are targetable with small molecule inhibitors.

## Supplementary information

Supplementary information

## References

[1] Butt NM, Lambert J, Ali S, Beer PA, Cross NC, Duncombe A (2017). Guideline for the investigation and management of eosinophilia. Br J Haematol.

[2] Swerdlow SH, Campo E, Harris NL, Jaffe ES, Pileri SA, Stein H (2017). WHO classification of tumours of haematopoietic and lymphoid tissues.

[3] Reiter A, Gotlib J (2017). Myeloid neoplasms with eosinophilia. Blood.

[4] Metzgeroth G, Schwaab J, Gosenca D, Fabarius A, Haferlach C, Hochhaus A (2013). Long-term follow-up of treatment with imatinib in eosinophilia-associated myeloid/lymphoid neoplasms with PDGFR rearrangements in blast phase. Leukemia.

[5] Cheah CY, Burbury K, Apperley JF, Huguet F, Pitini V, Gardembas M (2014). Patients with myeloid malignancies bearing PDGFRB fusion genes achieve durable long-term remissions with imatinib. Blood.

[6] Jawhar M, Naumann N, Knut M, Score J, Ghazzawi M, Schneider B (2017). Cytogenetically cryptic ZMYM2-FLT3 and DIAPH1-PDGFRB gene fusions in myeloid neoplasms with eosinophilia. Leukemia.

[7] Schwaab J, Knut M, Haferlach C, Metzgeroth G, Horny HP, Chase A (2015). Limited duration of complete remission on ruxolitinib in myeloid neoplasms with PCM1-JAK2 and BCR-JAK2 fusion genes. Ann Hematol.

[8] Khodadoust MS, Luo B, Medeiros BC, Johnson RC, Ewalt MD, Schalkwyk AS (2016). Clinical activity of ponatinib in a patient with FGFR1-rearranged mixed-phenotype acute leukemia. Leukemia.

[9] Schwaab J, Umbach R, Metzgeroth G, Naumann N, Jawhar M, Sotlar K (2015). KIT D816V and JAK2 V617F mutations are seen recurrently in hypereosinophilia of unknown significance. Am J Hematol.

[10] Wang SA, Tam W, Tsai AG, Arber DA, Hasserjian RP, Geyer JT (2016). Targeted next-generation sequencing identifies a subset of idiopathic hypereosinophilic syndrome with features similar to chronic eosinophilic leukemia, not otherwise specified. Mod Pathol.

[11] Pardanani A, Lasho T, Wassie E, Finke C, Zblewski D, Hanson CA (2016). Predictors of survival in WHO-defined hypereosinophilic syndrome and idiopathic hypereosinophilia and the role of next-generation sequencing. Leukemia.

[12] Valent P, Klion AD, Rosenwasser LJ, Arock M, Bochner BS, Butterfield JH (2012). ICON: eosinophil disorders. World Allergy Organ J.

[13] Dobin A, Davis CA, Schlesinger F, Drenkow J, Zaleski C, Jha S (2013). STAR: ultrafast universal RNA-seq aligner. Bioinformatics.

[14] Wang K, Li M, Hakonarson H (2010). ANNOVAR: functional annotation of genetic variants from high-throughput sequencing data. Nucleic Acids Res.

[15] Ye S, Dhillon S, Ke X, Collins AR, Day IN (2001). An efficient procedure for genotyping single nucleotide polymorphisms. Nucleic Acids Res.

[16] Rajala HL, Eldfors S, Kuusanmaki H, van Adrichem AJ, Olson T, Lagstrom S (2013). Discovery of somatic STAT5b mutations in large granular lymphocytic leukemia. Blood.

[17] Ma CA, Xi L, Cauff B, DeZure A, Freeman AF, Hambleton S (2017). Somatic STAT5b gain-of-function mutations in early onset nonclonal eosinophilia, urticaria, dermatitis, and diarrhea. Blood.

[18] Voskoboinik I, Thia MC, Trapani JA (2005). A functional analysis of the putative polymorphisms A91V and N252S and 22 missense perforin mutations associated with familial hemophagocytic lymphohistiocytosis. Blood.

[19] Walters DK, Mercher T, Gu TL, O’Hare T, Tyner JW, Loriaux M (2006). Activating alleles of JAK3 in acute megakaryoblastic leukemia. Cancer Cell.

[20] Da Costa L, Tchernia G, Gascard P, Lo A, Meerpohl J, Niemeyer C (2003). Nucleolar localization of RPS19 protein in normal cells and mislocalization due to mutations in the nucleolar localization signals in 2 Diamond–Blackfan anemia patients: potential insights into pathophysiology. Blood.

[21] Heath C, Cross NC (2004). Critical role of STAT5 activation in transformation mediated by ZNF198-FGFR1. J Biol Chem.

[22] Walz C, Ahmed W, Lazarides K, Betancur M, Patel N, Hennighausen L (2012). Essential role for Stat5a/b in myeloproliferative neoplasms induced by BCR-ABL1 and JAK2(V617F) in mice. Blood.

[23] Choudhary C, Brandts C, Schwable J, Tickenbrock L, Sargin B, Ueker A (2007). Activation mechanisms of STAT5 by oncogenic Flt3-ITD. Blood.

[24] Funakoshi-Tago M, Tago K, Abe M, Sonoda Y, Kasahara T (2010). STAT5 activation is critical for the transformation mediated by myeloproliferative disorder-associated JAK2 V617F mutant. J Biol Chem.

[25] Hantschel O, Warsch W, Eckelhart E, Kaupe I, Grebien F, Wagner KU (2012). BCR-ABL uncouples canonical JAK2-STAT5 signaling in chronic myeloid leukemia. Nat Chem Biol.

[26] Grimley PM, Dong F, Rui H (1999). Stat5a and Stat5b: fraternal twins of signal transduction and transcriptional activation. Cytokine Growth Factor Rev.

[27] Ariyoshi K, Nosaka T, Yamada K, Onishi M, Oka Y, Miyajima A (2000). Constitutive activation of STAT5 by a point mutation in the SH2 domain. J Biol Chem.

[28] Bandapalli OR, Schuessele S, Kunz JB, Rausch T, Stutz AM, Tal N (2014). The activating STAT5B N642H mutation is a common abnormality in pediatric T-cell acute lymphoblastic leukemia and confers a higher risk of relapse. Haematologica.

[29] Kiel MJ, Velusamy T, Rolland D, Sahasrabuddhe AA, Chung F, Bailey NG (2014). Integrated genomic sequencing reveals mutational landscape of T-cell prolymphocytic leukemia. Blood.

[30] Kucuk C, Jiang B, Hu X, Zhang W, Chan JK, Xiao W (2015). Activating mutations of STAT5B and STAT3 in lymphomas derived from gammadelta-T or NK cells. Nat Commun.

[31] Koskela HL, Eldfors S, Ellonen P, van Adrichem AJ, Kuusanmaki H, Andersson EI (2012). Somatic STAT3 mutations in large granular lymphocytic leukemia. N Engl J Med.

[32] Luo Q, Shen J, Yang Y, Tang H, Shi M, Liu J (2018). CSF3R T618I, ASXL1 G942 fs and STAT5B N642H trimutation co-contribute to a rare chronic neutrophilic leukaemia manifested by rapidly progressive leucocytosis, severe infections, persistent fever and deep venous thrombosis. Br J Haematol.

[33] Baer C, Muehlbacher V, Kern W, Haferlach C, Haferlach T (2018). Molecular genetic characterization of myeloid/lymphoid neoplasms associated with eosinophilia and rearrangement of PDGFRA, PDGFRB, FGFR1 or PCM1-JAK2. Haematologica.

[34] Babushok DV, Perdigones N, Perin JC, Olson TS, Ye W, Roth JJ (2015). Emergence of clonal hematopoiesis in the majority of patients with acquired aplastic anemia. Cancer Genet.

[35] Pham HTT, Maurer B, Prchal-Murphy M, Grausenburger R, Grundschober E, Javaheri T (2018). STAT5BN642H is a driver mutation for T cell neoplasia. J Clin Invest.

[36] Malcovati L, Karimi M, Papaemmanuil E, Ambaglio I, Jadersten M, Jansson M (2015). SF3B1 mutation identifies a distinct subset of myelodysplastic syndrome with ring sideroblasts. Blood.

[37] Papaemmanuil E, Gerstung M, Malcovati L, Tauro S, Gundem G, Van Loo P (2013). Clinical and biological implications of driver mutations in myelodysplastic syndromes. Blood.

[38] Jawhar M, Schwaab J, Schnittger S, Meggendorfer M, Pfirrmann M, Sotlar K (2016). Additional mutations in SRSF2, ASXL1 and/or RUNX1 identify a high-risk group of patients with KIT D816V(+) advanced systemic mastocytosis. Leukemia.

[39] Jeromin S, Haferlach T, Grossmann V, Alpermann T, Kowarsch A, Haferlach C (2013). High frequencies of SF3B1 and JAK2 mutations in refractory anemia with ring sideroblasts associated with marked thrombocytosis strengthen the assignment to the category of myelodysplastic/myeloproliferative neoplasms. Haematologica.

[40] Buitenhuis M, Baltus B, Lammers JW, Coffer PJ, Koenderman L (2003). Signal transducer and activator of transcription 5a (STAT5a) is required for eosinophil differentiation of human cord blood-derived CD34+ cells. Blood.

[41] Wingelhofer B, Maurer B, Heyes EC, Cumaraswamy AA, Berger-Becvar A, de Araujo ED (2018). Pharmacologic inhibition of STAT5 in acute myeloid leukemia. Leukemia.

