# Supplementary material for: Recurrent activating STAT5B N642H mutation in myeloid neoplasms with eosinophilia
Source: Leukemia. 2018 Dec 20;33(2):415–25. doi: 10.1038/s41375-018-0342-3 (PMC6365490; doi:10.1038/s41375-018-0342-3)
Supplement: Supplementary file 1 — Supplementary information [file 41375_2018_342_MOESM1_ESM.docx]

**SUPPLEMENTARY INFORMATION**

**Recurrent activating *STAT5B* N642H mutations in myeloid neoplasia**

**with eosinophilia**

**CONTENTS:**

Supplementary Figure 1. Sanger sequence confirmations of *STAT5B* N642H mutant cases

Supplementary Figure 2. *STAT5B* mutations are absent in cultured T-cells from positive cases

Supplementary Table 1: Additional mutations identified in *STAT5B* mutated cases


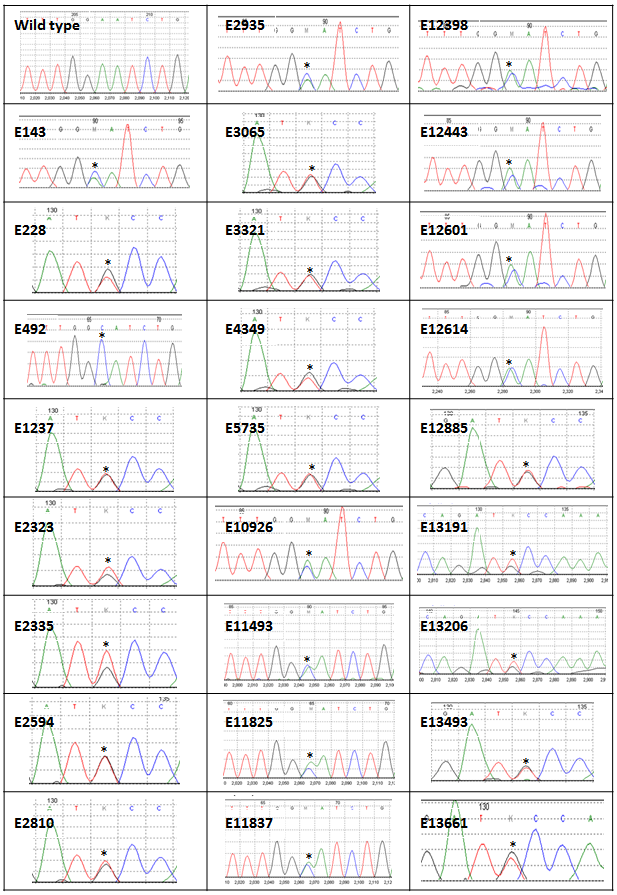


Supplementary Figure 1. Sanger sequence confirmations of *STAT5B* N642H mutant cases in either forward or reverse orientation. Mutations are indicated by asterisks.


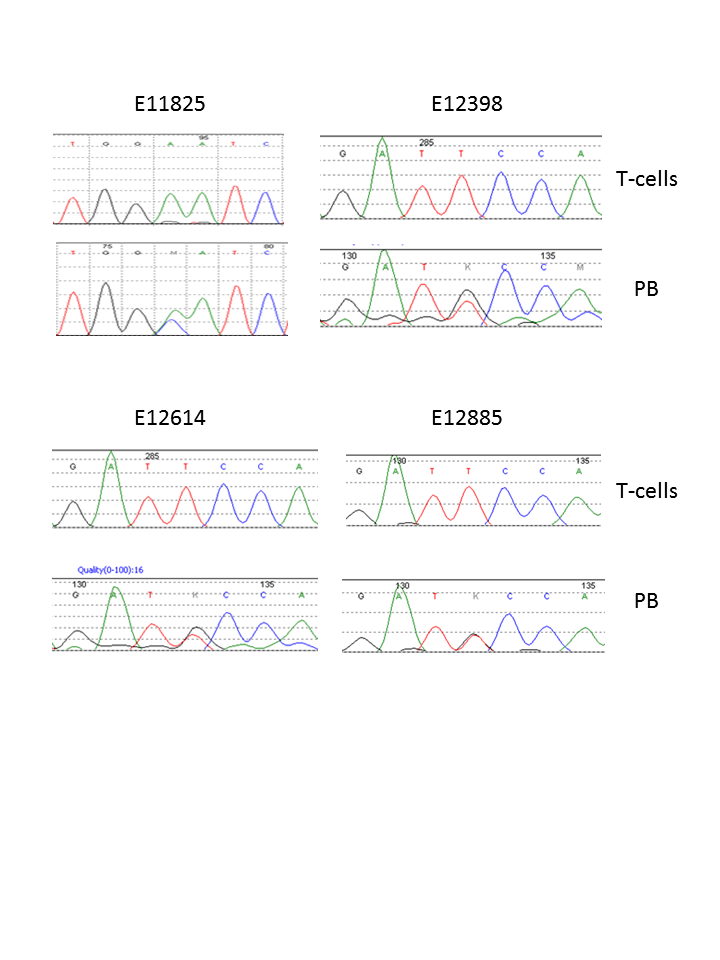
Supplementary Figure 2. *STAT5B* mutations are absent in cultured T-cells from cases that tested positive in total peripheral blood (PB) leucocytes.

| Case | Gene | Refseq | cDNA | Protein | vaf |
| --- | --- | --- | --- | --- | --- |
| E143 | *SF3B1* | NM_012433.3 | c.1998G>C | p.Lys666Asn | 0.47 |
|  | *TET2* | NM_001127208.2 | c.2830A>T | p.Lys944* | 0.46 |
|  | *TET2* | NM_001127208.2 | c.242C>T | p.Gln947* | 0.42 |
|  | *SRSF2* | NM_006265.2 | c.283C>G | p.Pro95Ala | 0.59 |
| E228 | *SF3B1* | NM_012433.3 | c.1998G>T | p.Lys666Asn | 0.46 |
| E492 | *CBL* | NM_005188.3 | c.1228-2A>G | Splice site | 0.19 |
| E1237 | *SRSF2* | NM_006265.2 | c.284C>A | p.Pro95His | 0.50 |
|  | *ASXL1* | NM_015338.5 | c.2075dupA | p.Arg693Alafs*25 | 0.44 |
| E2323 | *SRSF2* | NM_006265.2 | c.284C>G | p.Pro95Arg | 0.46 |
|  | *TET2* | NM_001127208.2 | c.439A>T | p.Lys147* | 0.46 |
|  | *TET2* | NM_001127208.2 | c.2111delA | p.Asn704Ilefs*47 | 0.48 |
|  | *NRAS* | NM_002524.3 | c.34G>C | p.Gly12Arg | 0.44 |
|  | *RUNX1* | NM_001754.4 | c.484A>G | p.Arg162Gly | 0.39 |
| E2335 | *U2AF1* | NM_006758.2 | c.470A>C | p.Gln157Pro | 0.40 |
|  | *ZRSR2* | NM_005089.3 | c.376C>T | p.Arg126* | 0.70 |
|  | *ASXL1* | NM_015338.5 | c.2893C>T | p.Arg965* | 0.42 |
|  | *RUNX1* | NM_001754.4 | c.806-1G>C | Splice site | 0.38 |
| E2594 | *SF3B1* | NM_012433.3 | c.2098A>G | p.Lys700Glu | 0.46 |
| E2810 | *SF3B1* | NM_012433.3 | c.1876A>C | p.Asn626His | 0.44 |
|  | *DNMT3A* | NM_175629.2 | c.1876A>C | p.Arg598* | 0.39 |
| E3321 | *SF3B1* | NM_012433.3 | c.1998G>C | p.Lys666Asn | 0.44 |
| E4349 | *DNMT3A* | NM_175629.2 | c.2173+1G>A | Splice site | 0.53 |
|  | *NRAS* | NM_002524.3 | c.35G>A | p.Gly12Asp | 0.05 |
|  | *EZH2* | NM_004456.4 | c.2233G>A | p.Glu745Lys | 0.45 |
|  | *RUNX1* | NM_001754.4 | c.351+1G>C | Splice site | 0.51 |
| E5735 | *SRSF2* | NM_006265.2 | c.284C>G | p.Pro95Arg | 0.45 |
|  | *TET2* | NM_001127208.2 | c.4664_4665delAG | p.Glu1555Valfs*22 | 0.45 |
| E10926 | *SF3B1* | NM_012433.3 | c.2098A>G | p.Lys700Glu | 0.46 |
|  | *TET2* | NM_001127208.2 | c.961C>T | p.Gln321* | 0.47 |
|  | *TET2* | NM_001127208.2 | c.2844delG | p.Lys948Asnfs*5 | 0.45 |
|  | *KIT* | NM_000222.2 | c.2447A>T | p.Asp816Val | 0.15 |
|  | *KRAS* | NM_033360.3 | c.40G>A | p.Val14Ile | 0.31 |
| E11076 | *SF3B1* | NM_012433.3 | c.1998G>T | p.Lys666Asn | 0.24 |
|  | *TET2* | NM_001127208.2 | c.1202delC | p.Pro401Hisfs*26 | 0.1 |
| E11493 | *SRSF2* | NM_006265.2 | c.284_307delCCCCGGACTCACACCACAGCCGCC | p.Pro95_Arg102del | 0.53 |
| E11825 | *SF3B1* | NM_012433.3 | c.2098A>G | p.Lys700Glu | 0.39 |
|  | *TET2* | NM_001127208.2 | c.507_508delTA | p.His169Glnfs*6 | 0.39 |
| E12398 | *ASXL1* | NM_015338.5 | c.1902_1924delAGAGGCGGCCACCACTGCCATCG | p.Glu635Argfs*15 | 0.14 |
|  | *CUX1* | NM_001202543.1 | c.3267T>A | p.Cys1089* | 0.52 |
| E12433 | *SF3B1* | NM_012433.3 | c.2098A>G | p.Lys700Glu | 0.45 |
| E12601 | *SRSF2* | NM_006265.2 | c.284C>A | p.Pro95His | 0.46 |
|  | *IDH2* | NM_002168.3 | c.419G>A | p.Arg140Gln | 0.50 |
|  | *CBL* | NM_005188.3 | c.1255T>C | p.Cys419Arg | 0.25 |
|  | *CBL* | NM_005188.3 | c.1259G>A | p.Arg420Gln | 0.07 |
| E12885 | *U2AF1* | NM_006758.2 | c.470A>C | p.Gln157Pro | 0.51 |
|  | *ASXL1* | NM_015338.5 | c.1924G>T | p.Gly642* | 0.26 |
| E13191 | *EZH2* | NM_004456.4 | c.2069G>A | p.Arg690His | 0.94 |
| E13206 | *EZH2* | NM_004456.4 | c.728delA | p.Lys243Asnfs*22 | 0.36 |
| E13493 | *TET2* | NM_001127208.2 | c.3250C>T | p.Gln1084* | 0.46 |
|  | *EZH2* | NM_004456.4 | c.1978G>A | p.Gly660Arg | 0.47 |
|  | *ASXL1* | NM_006758.2 | c.1934dupG | p.Gly646Trpfs*12 | 0.40 |
| E13661 | *JAK2* | NM_001322194.1 | c.1849G>T | p.Val617Phe | 0.55 |
|  | *IDH2* | NM_002168.3 | c.419G>A | p.Arg140Gln | 0.46 |

Supplementary Table 1: Additional mutations identified in *STAT5B* mutated cases
